# Application of Monte Carlo simulation and artificial neural network model to probabilistic health risk assessment in fluoride-endemic areas

**DOI:** 10.1016/j.heliyon.2024.e40887

**Published:** 2024-12-04

**Authors:** Raisul Islam, Alok Sinha, Athar Hussain, Mohammad Usama, Shahjad Ali, Salman Ahmed, Abdul Gani, Najmaldin Ezaldin Hassan, Ali Akbar Mohammadi, Kamlesh Deshmukh

**Affiliations:** a Department of Civil Engineering, GLA University Mathura, India; b Department of Environmental Science and Engineering, IIT, (ISM), Dhanbad, Jharkhand, India; c Department of Civil Engineering, Netaji Subhas University of Technology, New Delhi, India; d Department of Environmental Science, Integral University, Lucknow, India; e Department of Environmental Science, Sharda School of Smart Agriculture, Sharda University Agra, Keetham, Agra, 282007, India; f Interdisciplinary Department of Remote Sensing and GIS Applications, Aligarh Muslim University, Aligarh, India; g Department of Civil and Environment Engineering, University of Zakho, Kurdistan Region, Iraq; h Department of Environmental Health Engineering, Neyshabur University of Medical Sciences, Neyshabur, Iran; i Department of Computer Science and Engineering, Anand Engineering College, Agra, India; j Workplace health research center, Neyshabur University of Medical Sciences, Neyshabur, Iran

**Keywords:** Artificial neuro network, Monte Carlo simulation, Probabilistic health risk assessment, Fluorosis, Kasganj area

## Abstract

Groundwater contamination with fluoride is a considerable public health concern that affects millions of people worldwide. The rapid growth of urbanization has led to increase in groundwater contamination. The health risk assessment focuses on both acute and chronic health consequences as it investigates the extent and effects of fluoride exposure through contaminated groundwater. Fluoride exposure, especially in endemic locations, has serious health consequences, including dental and skeletal fluorosis. An accurate assessment of these hazards is essential for public health planning and mitigation actions. The present study uses Monte Carlo Simulation (MCS) and an Artificial Neural Network (ANN) model to perform a Probabilistic Health Risk Assessment on populations in fluoride-endemic areas. Analysis of the results of the study reveals that the concentration of fluoride ranged from 0.58 to 3.80 mg/L with an average of 2.30 mg/L across the Kasganj district, which was higher than permissible limits given by BIS and WHO. The highest value of hazard quotient of 3.29 for Children is found to be in the Durga Colony area, while the lowest value of the hazard quotient of 0.31 for adults is found to be in the Nadrai Gate area. The assessment of health risks revealed a high probability of non-carcinogenic disease from the consumption of groundwater containing fluoride. The ANN model has the R^2^ value of 0.9989 in training and 0.9870 in testing while RMSE value in training and testing was 0.02230 and 0.0267. The findings suggest that before being used, the groundwater in Kasganj, Uttar Pradesh, India, needs to be treated and made drinkable. The results emphasize the critical need for ongoing monitoring, public education initiatives, and implementing feasible mitigating techniques to lower fluoride exposure. The findings show that this hybrid model is excellent at addressing the numerous uncertainties associated with fluoride use, hence improving the reliability of health risk estimates in fluoride-endemic locations. The results offer vital information to help policymakers and local health officials create focused measures to safeguard public health in Kasganj.

## 1 Introduction

Due to the increasing urbanization of developing countries, robust economic growth, and the quick expansion of businesses like refineries and heavy automotive production, environmental pollution is becoming a pressing global issue [1]. Groundwater (GW) is the main source of potable water for most modern-day urban areas, farms, and businesses; however, its quality is progressively decreasing due to population growth and a host of industrial processes [1]. Arid and semi-arid regions, especially in developing countries like India and Bangladesh, see a dramatic spike in water demand as a result of fast population growth brought about by intense development activities [2,3]. Fluoride is recognized as a trace element that is essential for the growth and formation of teeth and bones [4]. Potable water contains fluorides naturally at low concentrations are good for teeth. However, high concentrations of fluoride in drinking water can cause skeletal, dental and bone fluorosis, depending on the amount consumed and the length of time exposed [5]. Fluoride (F^−^) levels in drinking water are presently a major issue.

Although fluoride concentration of 1.5 mg/L is deemed acceptable, levels higher than that pose health risks to humans [6]. In areas where there are high-risk factors for the development of dental caries, such as areas with a very high sugar intake, fluoride is used to prevent caries from occurring. Fluoride is additionally found in fish, tea, formula milk, salt, fluoridated toothpaste, mouthwash, and varnish, although it is most commonly associated with water fluoridation [7]. Approximately 200 million people in 25 countries are consuming water that is contaminated with fluoride [[8], [9], [10], [11]]. The dissolution of granitic rocks that are enriched with fluoride, whether occurring naturally or due to human activity, can result in a significant concentration of fluoride in groundwater. Minerals such as biotite, cryolite, fluorapatite, fluorite, hornblende, muscovite, and topaz, which are abundant in fluorine, can be found in certain rocks. Some rocks readily dissolve in groundwater and release fluoride ions [12,13]. The rise in F^−^ ions in GW is attributable, in part, to human activities such as burning coal, producing industrial waste, and using too much fertilizer on agricultural land. In geology, fluoride can have several origins, such as ion exchange, changes in rock-water morphology, rock composition, and calcite precipitation [[14], [15], [16]].

Prolonged exposure to concentrations of fluoride exceeding 1.5 mg/L leads to significant health complications such as fluorosis, which includes dental, bone, and skeletal fluorosis, as well as bone cancer and impotence [4,[17], [18], [19], [20], [21]].

Therefore, the estimation of human health risks is essential to become aware of the associated potential fluoride hazard with water contamination. Monte Carlo Simulation (MCS) is a strong probabilistic tool that allows for the evaluation of health risks by modelling a large range of possible exposure scenarios using probability distributions of important variables. MCS has demonstrated effectiveness in generating more realistic risk estimations by accounting for variability in parameters such as fluoride content and individual intake rates. While MCS captures uncertainty, it may not always accurately represent the complicated, non-linear connections between numerous exposure variables and health consequences [22].

Traditional techniques to fluoride risk assessment frequently rely on deterministic methodologies that use pre-set values for exposure parameters such as fluoride concentration, water intake, and body weight. However, these methodologies may fail to account for the inherent unpredictability and uncertainty in environmental and human factors, thus leading to erroneous risk assessments. To address these constraints, probabilistic risk assessment approaches are increasingly being adopted, which allow for a more comprehensive risk evaluation by factoring in uncertainty and variability [22,23].

There is still a lot of unsolved questions about using ANN to forecast the hazard quotient (HQ) because of fluoride pollution, even though ANN has great promise. More complex modeling approaches, such as ANN, have not been thoroughly investigated in the literature, with most studies concentrating on linear or less complex nonlinear models. ANN models are being used to estimate groundwater quality, but their usage to predict fluoride contamination's HQ is understudied. For analyzing fluoride's non-carcinogenic health effects, the HQ must accurately estimate health risk. ANN models have been shown to predict fluoride levels, although HQ prediction remains understudied [23]. MCS has demonstrated effectiveness in generating more realistic risk estimations by accounting for variability in parameters such as fluoride content and individual intake rates. While MCS captures uncertainty, it may not always accurately represent the complicated, non-linear connections between numerous exposure variables and health consequences [24,25]. To address this issue, the use of ANN presents a fresh method to risk modelling. ANNs can recognise and predict complicated patterns in data, making them ideal for capturing the nonlinear relationships between fluoride exposure parameters and the likelihood of health effects. Combining MCS and ANN creates a powerful tool for Probabilistic Health Risk Assessment (PHRA), allowing for more accurate and thorough study of fluoride-related risks in endemic areas.

This gap allows researchers to design and evaluate ANN-based models that accurately estimate HQ, improving fluoride contamination management and public health [25].

The main objectives of current research work are as follows: (a) Collection of water samples and check for the fluoride concentration and compare them to both national and international benchmarks (b) Determine the potential dangers of fluoride exposure from polluted drinking water for three distinct demographics (children, teenagers, and adults) using deterministic and probabilistic techniques (c) using the Geospatial Analysis software to describe the spatial distribution of fluoride in drinking water in study area (d) To predict the health risk by the fluoride concentration with the help of ANN in Kasganj district, Uttar Pradesh. The results of this study will be useful in determining the best course of action, which may include providing access to centralized and alternative water supply sources.

## 2 Methods and materials

**Fig. 1:**
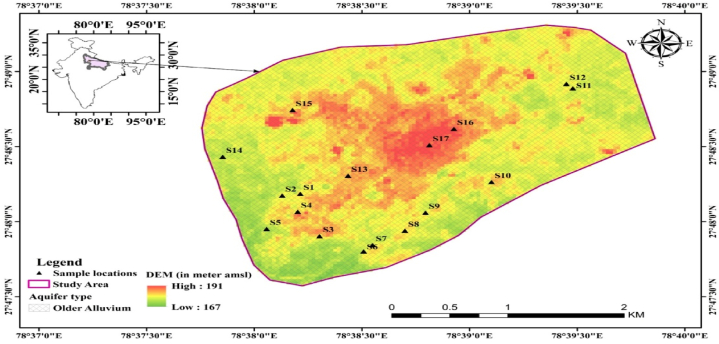
Locations of Kasganj, Uttar Pradesh, northern India.

The present research was focused on area of Kasganj, Uttar Pradesh, India. The region possesses a notable geographical feature in the form of the Kali River, Uttar Pradesh, India, between 27°47′ N and 78°39′ E (Fig. 1).

The samples were collected from the targeted area using wells, tube wells, submersibles and hand pumps. The collection of all samples occurred within a year, specifically from May 2023 to April 2024. 85 groundwater samples had been preserved in polypropylene bottles at 4 °C after being collected from 17 locations (5 by 10). Using ArcGIS 10.2, the sampling locations have been plotted (Fig. 1). Collection of samples was done after the wells, tube wells, submersibles and hand pumps were used for some time so that physio-chemical parameters became stable. The concentration of fluoride were studied using the UV-1800 spectrophotometer Shimadzu [26].

### 2.1 Evaluation of the fluoride risk to human health via exposure analysis

The health risk due to fluoride in GW was estimated using the US Environmental Protection Agency's (USEPA) 1989 model. Fluoride contaminant in potable water, were evaluated for their non-carcinogenic potential using this model. It is based on Eq. (1). Table 1 lists the characteristics that were utilized to calculate the chronic daily intake (CDI) by using Equation (1) [22,27].

Eq. (1) $$CDI=\frac{C*\text{IR}*\text{EF}*\text{ED}}{\text{BW}*\text{AT}}$$

Eq. (2) $$HQ=\frac{CDI}{Rfd}$$

**Table 1:** Parameters used for health risk assessment using MCS and uncertainty analysis [5,[28], [29], [30]].

Parameters	Adults (18–62)	Teenagers (7–18)	Children (2–6)	Unit
Average time (AT)	14600	4745	1460	days
Exposure frequency (EF)	365	365	365	days/year
Bodyweight (BW)	78	50	15	Kg
Ingestion rate (IR)	2.5	2	0.78	L/d
Reference dose (RfD)	0.06	0.06	0.06	mg/kg/day
Exposure duration (ED)	40	13	4	Year
C″	2.3	2.3	2.3	

HQ is used in Equation (2) to calculate the non-carcinogenic risk associated with exposure of fluoride concentration [5,22,31].

The reference dosage (RfD), utilized for risk assessment calculations, estimates the daily intake of a population without a substantial risk of harmful consequences over a period of time.

The RfD values for fluoride (0.06 mg/kg/d) was obtained in the database of the Integrated Risk Information System. Equation (2) is utilized for the computation of the Hazard Quotient. Elevated levels of concentration, over the threshold, are likely to have adverse effects on disease and health. HQ > 1 indicates a non-carcinogenic risk [22,28,32].

### 2.2 Sensitivity analysis (SA) and Monte Carlo simulation (MCS)

To calculate the human health risk, MCS was used to account for uncertainty and variability in several parameters (Table 1). In order to do the sensitivity analysis, 10,000 iterations were run using the Oracle Crystal Ball programme for MCS, version 11.1.34190. From their fitted distribution, MCS selected the parameter values that allowed them to assess exposure risk and point value [33]. Variations in MCS output, which might be attributed to changes in the input data, were examined using SA [34]. The probability distribution functions utilized by the MCS and SA were evaluated by the USEPA [27].

### 2.3 Development of the ANN model

**Fig. 2:**
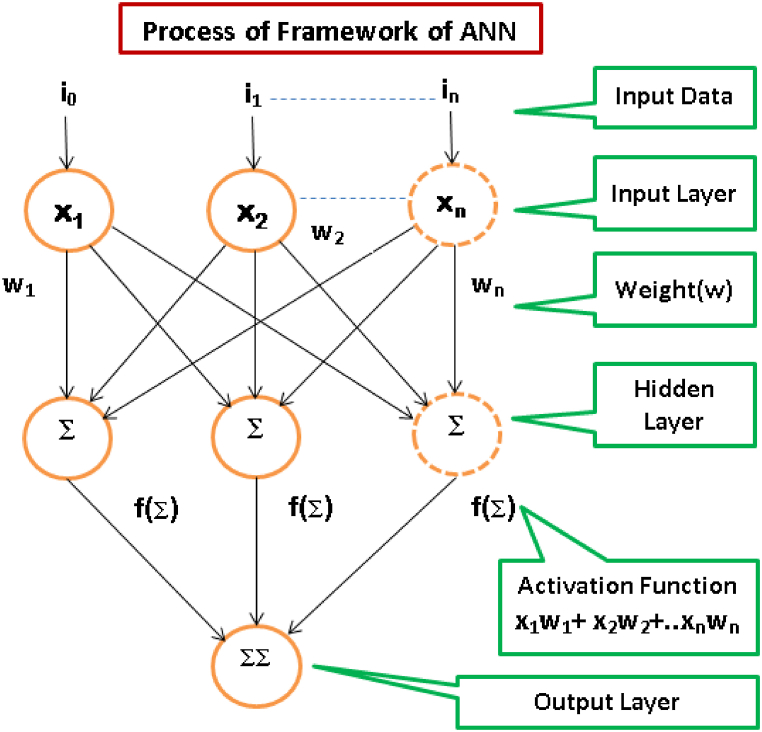
Process of framework of ANN model.

While designing and developing complex systems, it may be rigorous to set the correlation of target variables in accordance with the inputs. In such situations, ANN proffer an exclusive and profound resolution to address the issues. Predictions are the widely popular uses for this technology [35]. The significant stepladders in designing and developing ANN models are essential to choosing applicable inputs, selecting of structure of the network, pre-processing and splitting the available input data, selecting a network architecture, laying down performance standards, training, testing and validation [36,37]. Fig. 2 shows the procedure of the ANN framework.

### 2.4 Optimization of network architecture

As compared to creation of a mathematical model based on non-linear regression is in general applied to the accurate prediction of HQ by applying a mathematical function. Fluoride concentration, IR, EF, ED, BW, and AT are used to evaluate CDI. To calculate the HQ, CDI value is divided by a constant value of RfD. A well-connected, parallel equipped with feed-forwarding, a more sophisticated neural network-based regression model instead of simple linear regression analysis was used. The two primary steps in building this model were choosing the network architecture and establishing the network's structure.

The initial stage focuses on the Input Layer that accepts the feature set and consists of six dimensions (fluoride, EF, IF, ED, BW, and AT), hidden layers with several configurations equipped with Rectified Linear Unit (ReLU) activation function and L2 regularization, and at last the output layer equipped with “Linear” activation function target to HQ. By applying different configurations of hidden layers, learning rates, and regularization strengths are tested using 5-fold cross-validation. However, the second stage determines the parameters used for training that are based on the best configuration, and early stopping is applied to prevent overfitting. The complete training set is used to train the model, and the test set is used to evaluate it together with the number of iterations. Along with training ending conditions, it also contains the learning rate and a training algorithm that goes through several retrains [38].

This ANN model implementation determines a thorough line of attack to building, tuning, and evaluating a best multi-layered and 5 version regression model for predicting the HQ based on fluoride concentration. The use of standard machine learning techniques such as data standardization, k-fold cross-validation, Hyperparameter tuning, regularization, and early stopping ensures that the model is both accurate and generalizable [39].

In the present study, 85 samples have been available for carrying out modeling with 6 input parameters (neurons). These 85 samples were split into three groups: (i) training, (ii) K-fold cross-validation, and (iii) testing. In the present study, the model contains multiple hidden layers whose best configuration is determined through Hyperparameter tuning where each hidden layer comprises of a definite number of neurons, and the number of neurons in each layer can vary. These configurations tested include: Three hidden layers with 64, 32, and 16 neurons respectively, three hidden layers with 128, 64, and 32 neurons respectively, two hidden layers with 64 and 32 neurons respectively, and two hidden layers with 128 and 64 neurons respectively. Each hidden layer uses the ReLU activation function, which introduces non-linearity to the model, and L2 regularization was applied to each hidden layer to prevent over fitting, with values tested including 0.01 and 0.1.

**Fig. 3:**
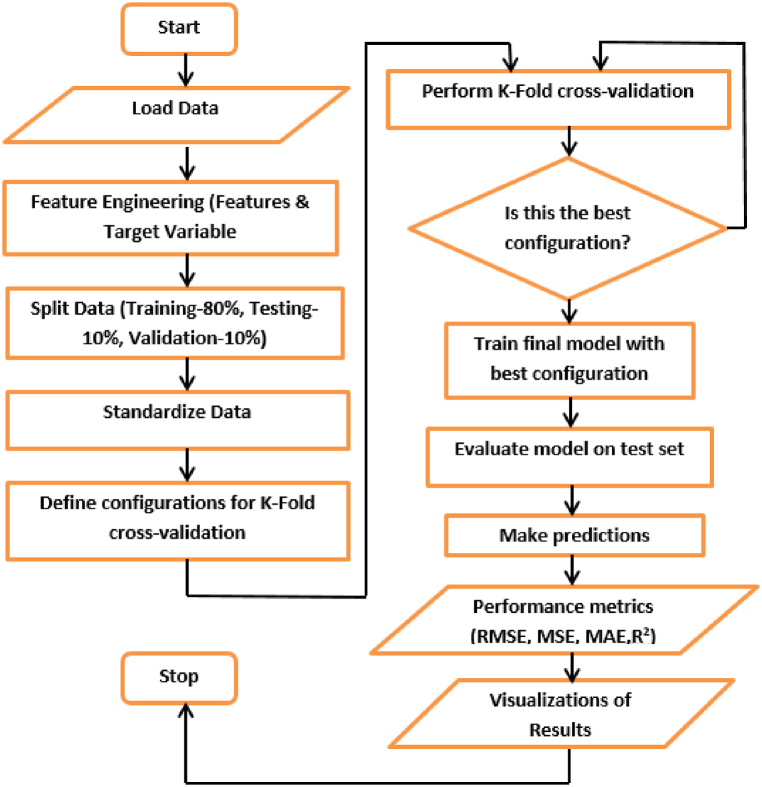
Flow Diagram of ANN algorithm.

The ideal framework for included fragmentation was 80%–10%–10 %. It consists of dataset distributions with 80 %, 10 %, and 10 % of each going to the training, cross-validation, and testing datasets, respectively. The HQ predictions produced by the aforementioned network have a significant, very elevated, strong correlation with measured HQ scores (r = 1, p = 0.01), indicating that these estimates explain nearly 99.85 % of the various variants in the traditionally determined HQ values. The network's training error mean squared error (MSE) was 0.000579. The process flow of ANN algorithm is depicted in Fig. 3.

### 2.5 Assessment of performance and selection of models

The training dataset has been used to estimate neural network parameters for various specifications. After testing the trained network on the validation set, the prediction model with the best performance is chosen [40]. The testing set is used to evaluate the model's validity, usefulness, and generalization performance using a performance metric such mean square error (MSE). This method uses the coefficient of determination (R^2^) to evaluate how well the model fits the data and predicts it. Thus, the best R^2^ value may be used to choose the best model from numerous [41]. In this work, MSE and linear regression analysis were used to evaluate the proposed model's HQ prediction ability. Present research work compares prospective ANN models based on cross-validation and testing set errors, correlations between projected and reported HQ values, and the model's ability to predict observed HQ value change. In the present study, the ANN model was built with the lowest cross-validation and subset deviation testing with excellent agreement.

## 3 Result and discussion

### 3.1 Spatial distribution of fluoride in kasganj, U. P., India

**Fig. 4:**
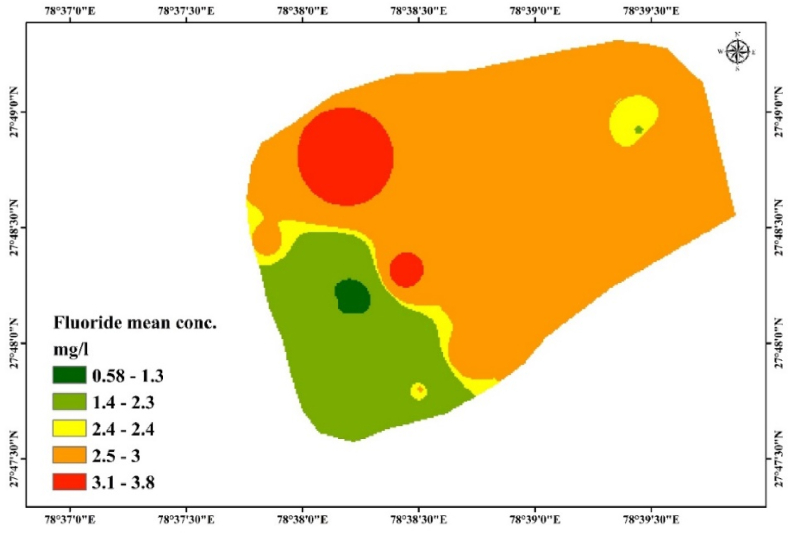
Distribution of F^−^ concentration in Kasganj, U. P, India.

The concentration of fluoride in drinking water exceeds 1.5 mg/L, causing dental and skeletal fluorosis [[42], [43], [44]]. The fluoride means concentrations in the water of Kasganj ranged from 0.58 to 3.8 mg/L, with an average mean of 2.30 mg/L, as shown in Table 2. The groundwater sample from Durga Colony had the highest level of fluoride at 3.80 mg/L, while the one from Nadrai Gate had the lowest at 0.58 mg/L. About 88 % of the samples (Table 2 and Fig. 4), respectively, were found to be above the surpassed limit of 1.5 mg/L (BIS 2012) Indian Standards and WHO [42,43,45]. Five samples were collected from every spot in the study area, and the spatial distribution of fluoride is shown in Fig. 4. On average, these five points formed the basis for the zoning computations, Monte Carlo simulations, and risk assessment evaluations. Numerous samples failed to fulfill the criteria set by the World Health Organization, as seen in the aforementioned report. A study conducted by Salman et al. (2020), area of Mathura, U.P, India found the elevated level of concentration of fluoride from 0.03 to 1.71 [46].As reported by Yadav et al. (2019), Agra City, U. P, India has an increased range of levels of fluoride between 0.41 and 3.99 [6]. A research work investigated by Ali et al., in 2017 found that certain blocks in the Agra district had higher groundwater fluoride concentrations than others, with values ranging from 0.14 to 4.88 mg/L [4]. In Unnao, Uttar Pradesh, India, Ansari and Umar (2019), conducted a one more research that produced remarkably similar findings. They discovered that the concentration of fluoride ions ranged from 0.06 to 1.83 mg/L in different blocks of Unnao, U. P., India [47]. In Varanasi, U. P., India, fluoride concentrations ranged from 0.28 to 2.01, according to a very comparable study conducted by Chaurisiaya et al. (2018) [48]. In Banda, U.P., India. Singh et al. (2016) investigated the research work that the concentration of fluoride ions ranges from 0.32 to 3.5 mg/L [49]. Dev and Raju (2014), revealed that level of F^−^ from 0.08 to 6.7 in Sonbadra, U. P., India [50]. Therefore, a significant area of northern India is endemic to high fluoride concentrations (Table 3).

**Table 2:** The minimum, maximum and mean level of fluoride in Kasganj, U. P, India.

S. No.	Location	Min	Max	Mean	STV
S1	Nadrai Gate	0.50	0.70	0.58	0.08
S2	Madhopuri Colony	1.32	1.48	1.40	0.06
S3	Maharana Pratap Colony	1.72	1.90	1.80	0.06
S4	PWD Colony	1.65	1.90	1.80	0.09
S5	Officer Colony	1.70	1.88	1.80	0.07
S6	Sahawar Gate	1.88	2.80	2.40	0.35
S7	Railway Road	1.65	1.90	1.80	0.09
S8	Ahrauli	2.40	2.90	2.60	0.23
S9	Arya Nagar	1.80	3.10	2.50	0.54
S10	Amapur Road	2.30	3.10	2.78	0.29
S11	Jakharrudder pur	2.30	2.80	2.50	0.21
S12	Awas Vikas Colony	1.10	2.80	2.24	0.72
S13	Tarora	2.90	3.90	3.40	0.37
S14	Bilram Gate	1.60	3.00	2.42	0.51
S15	Durga Colony	3.60	4.00	3.80	0.16
S16	Yadav Nagar	2.20	3.10	2.65	0.38
S17	Jay Jay Ram Mohalla	2.50	2.80	2.66	0.11

**Table 3:** Fluoride content in the groundwater of U. P., India [4,6,[46], [47], [48], [49], [50], [51]].

Location of U.P., India	Fluoride level in mg/L	Fluoride Concentration sources
Kasganj (Current Study)	0.58 to 3.80	Geogenic formation and anthropogenic sources
Mathura	0.03 to 1.71	The dominance of weathering of rock forming minerals, with secondary contributions from manmade sources.
Agra	0.90 to 4.12	Geogenic formation and anthropogenic sources
Agra	0.14 to 4.88	Rocks with fluorine minerals that interact with groundwater.
Unnao	0.06 to 1.83	Due to agricultural activity and Brick ash
Varanasi	0.28 to 2.01	Geogenic
Banda	0.32 to 3.5	From F^−^ minerals
Sonbhadra	0.08 to 6.7	Occurring due to natural causes mainly due to geogenic-water interaction

### 3.2 Assessment of exposure

Total daily intake (TDI) and CDI of fluoride through oral ingestion were calculated for exposure assessment. For the seventeen numerous locations in Kasganj, U.P., India, CDI values have been estimated for infants, children and adults. The chronic daily consumption for targeted groups were determined independently based on the fluoride concentration reported in Table 4. The exposure dose varied widely among children (0.45–2.96), teenagers (1.16–7.60), and adults (1.45–9.50). Among the various age groups investigated, the exposure dose in children, teenagers, and adults were 1.97, 1.90, and 2.37 times greater than the adequate and safe daily fluoride intakes advised by NRC [4,52]. This means that adults consume 1.20 times more fluoride than children and 1.5 times as much as teenagers.

**Table 4:** CDI values for targeted groups in Kasganj, U. P., India.

S.No.	Children (2–6)			Teenagers (7–18)			Adults (18–62)		
	CDI_min_	CDI_max_	CDI_avg_	CDI_min_	CDI_max_	CDI_avg_	CDI_min_	CDI_max_	CDI_avg_
S1	0.390	0.546	0.452	1.000	1.400	1.160	1.250	1.750	1.450
S2	1.030	1.154	1.092	2.640	2.960	2.800	3.300	3.700	3.500
S3	1.342	1.482	1.404	3.440	3.800	3.600	4.300	4.750	4.500
S4	1.287	1.482	1.404	3.300	3.800	3.600	4.125	4.750	4.500
S5	1.326	1.466	1.404	3.400	3.760	3.600	4.250	4.700	4.500
S6	1.466	2.184	1.872	3.760	5.600	4.800	4.700	7.000	6.000
S7	1.287	1.482	1.404	3.300	3.800	3.600	4.125	4.750	4.500
S8	1.872	2.262	2.028	4.800	5.800	5.200	6.000	7.250	6.500
S9	1.404	2.418	1.950	3.600	6.200	5.000	4.500	7.750	6.250
S10	1.794	2.418	2.168	4.600	6.200	5.560	5.750	7.750	6.950
S11	1.794	2.184	1.950	4.600	5.600	5.000	5.750	7.000	6.250
S12	0.858	2.184	1.747	2.200	5.600	4.480	2.750	7.000	5.600
S13	2.262	3.042	2.652	5.800	7.800	6.800	7.250	9.750	8.500
S14	1.248	2.340	1.888	3.200	6.000	4.840	4.000	7.500	6.050
S15	2.808	3.120	2.964	7.200	8.000	7.600	9.000	10.000	9.500
S16	1.716	2.418	2.067	4.400	6.200	5.300	5.500	7.750	6.625
S17	1.950	2.184	2.075	5.000	5.600	5.320	6.250	7.000	6.650

### 3.3 Hazard characterization

According to Naz et al. (2016) [53], there was minimal individual risk for both children and adults since the HQ of all components, calculated using mean concentration, were less than 1. Ingesting fluoride can cause a variety of negative health effects, including nausea, neurotoxicity, and even death [54]. The most prevalent consequence of chronic fluoride salt consumption is the effect that is of concern in our risk evaluation. There are three potential sites where fluoride non-carcinogenic effects could manifest: the teeth, bones, and skeleton.

### 3.4 Dental fluorosis

**Fig. 5:**
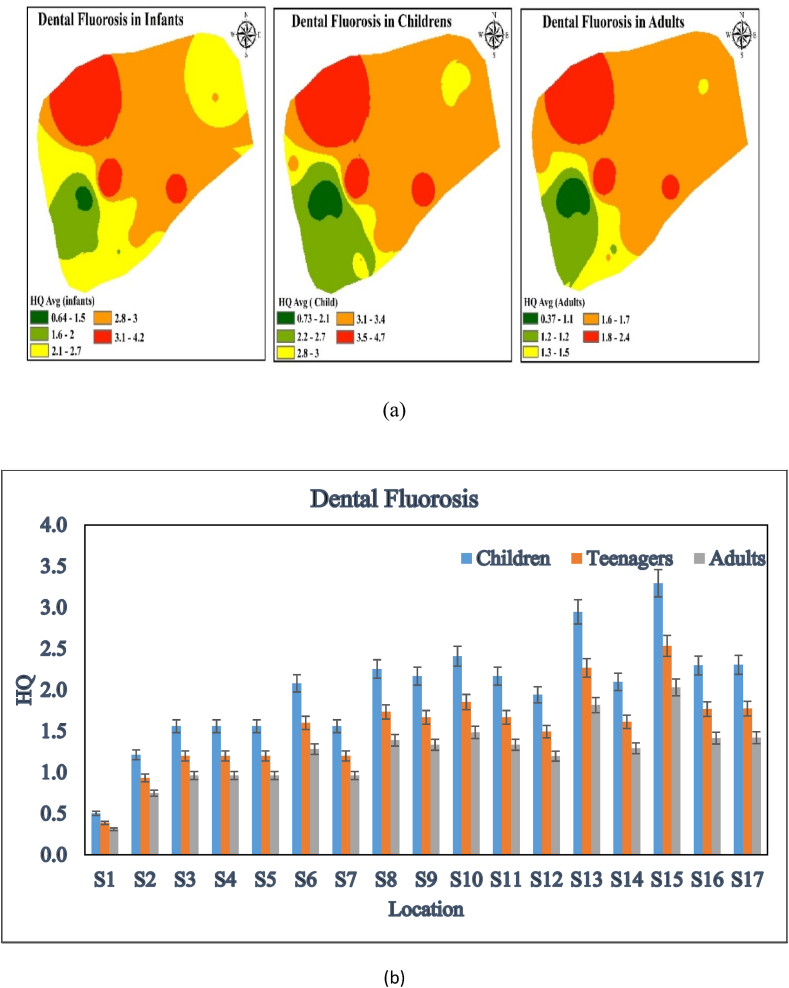
a, b HQ level in different locations for dental fluorosis in Kasganj, U.P., India.

Dental fluorosis, which happens when children are exposed to fluoride while their enamel is forming, can discolor and destroy teeth. Fluoride in drinking water above 1.0 mg/L puts people, especially children, at risk of fluorosis. The breakdown of metabolic calcium and phosphorus and reduction of human enzymatic activity impair the endocrine system, causing fluorosis [4]. The sampled location had the highest non-carcinogenic risk, suggesting dental fluorosis was more likely. Fig. 5 a, b shows that children had the highest risk (0.725–4.75), followed by infants (0.64–4.22) and adults (0.37–2.44). Children (1.95), higher non-carcinogenic risk than adults. Milk, which is vital for growing bodies, may have more fluoride than formula because evaporation reduces the volume of hot water used to reconstitute milk. Children may have higher HQ values than adults because to their lower body weight. At 2–6 years old, this hazardous disease is more prevalent than at 18 [5,21,55]. Dental fluorosis is typically determined by fluoride exposure up to age 8–10, as it stains developing teeth in the jawbones and under the gums. If adults' teeth have fully formed before fluoride exposure, dental fluorosis may not be noticeable. If individuals do not exhibit dental fluorosis, their fluoride consumption may not be under the safe range [56].

### 3.5 Bone fluorosis

**Fig. 6:**
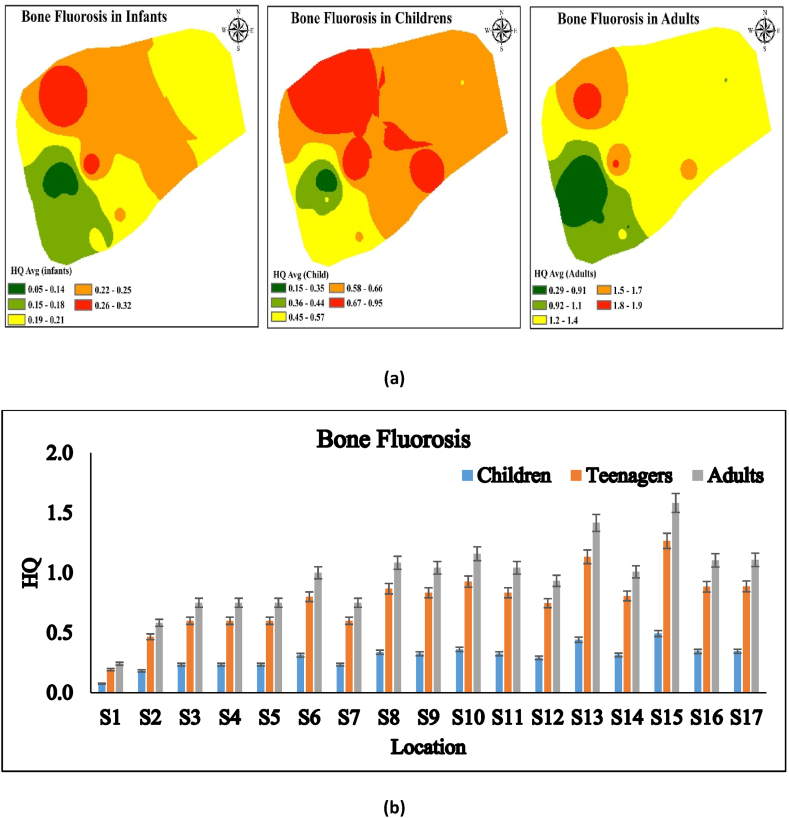
a, b HQ level in different locations for bone fluorosis in Kasganj, U.P., India.

HQ values ranged from 0.290 to 1.90 in Kasganj, U.P., India, with Durga colony having the highest HQ (1.90) (Fig. 6a and b). Adults were more affected by bone fluorosis than children and infants. Only skeletal fluorosis begins with bone fluorosis. Studies demonstrate that 99 % of an individual's fluoride intake is retained in bones. Fluoride intake alters bone tissue reabsorption and accretion, affecting bone mineral metabolism. Although age, sex, and bone type affect bone fluoride content, the accumulation rate declines with age and achieves equilibrium after 50 years [4,57].

### 3.6 Skeletal fluorosis

**Fig. 7:**
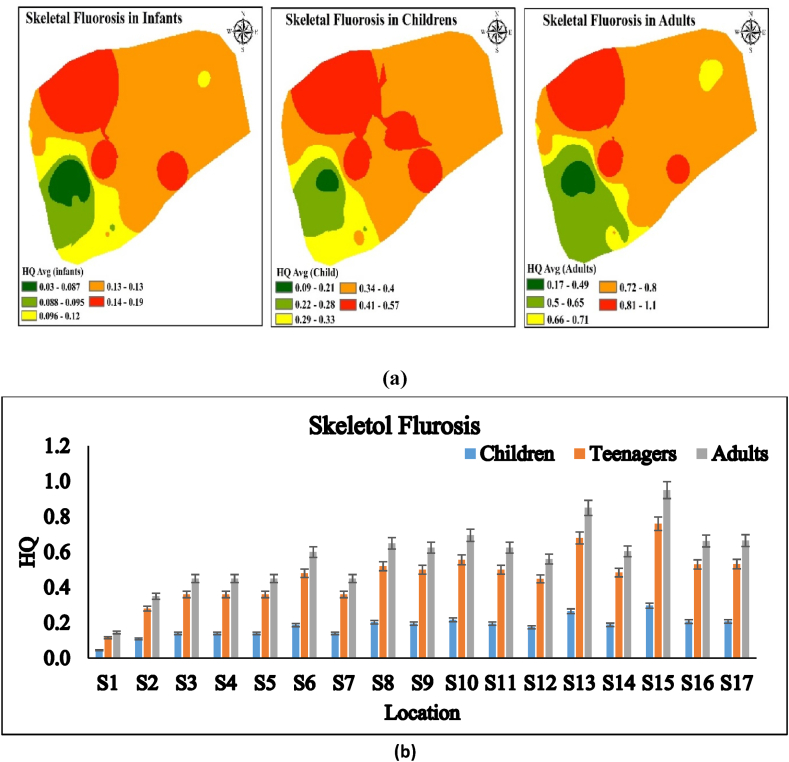
a, b HQ level in different locations for skeletal fluorosis in Kasganj, U.P., India.

Skeletal fluorosis is an advanced stage of bone fluorosis that causes discomfort and damage to bones and joints owing to excessive fluoride accumulation. Similar location-wise HQ values were found for dental and bone fluorosis. Age-wise, adults showed the highest HQ values (1.14), while infants had the lowest (0.029) (Fig. 7a and b). Thus, infants are immune to skeletal fluorosis while adults are more susceptible. Choubisa (2001) found that skeletal fluorosis prevalence rises with age and fluoride concentration [58]. According to Krishnamachari (1986), the amount of fluoride ingested strongly impacts the clinical course of skeletal fluorosis, additionally, the severity of symptoms correlates with the level and duration of exposure [59,60]. The World Health Organization (1984) states that in order to acquire severe skeletal fluorosis, one needs consume 20–80 mg/day of fluoride daily, which is the same as 10 mg/L of fluoridated water [4,61]. the current work indicated that 11.40 mg/day was the upper limit for oral consumption. In the research area, debilitating skeletal fluorosis is unlikely. High fluoride intake may cause severe skeletal fluorosis in the future. The present research work revealed that adults are more likely to be impacted by fluoride-contaminated water than children and infants due to their increased exposure.

### 3.7 Evaluating and comparing the occurrence of skeletal, bone, and dental fluorosis

The study revealed that of three types of fluorosis i.e., dental, bone and skeletal, children are more prone to dental fluorosis (94 %) followed by teenagers (88 %) showed in Table 6. Ali et al. (2017) showed that the probability of dental fluorosis in infants was higher (42 %) while adults were more prone to bone and skeletal fluorosis (60 %) [4]. The prevalence of dental fluorosis was found to be 45.7 % in southern Rajasthan (Choubisa, 2001) and 35 % in Karnataka (Bharati et al., 2005), correspondingly [58,62]. In dental fluorosis, fluoride-induced enamel lesions interrupt enamel growth and hypo mineralize the enamel. As shown in Table 5, adults are more prone to bone fluorosis (59 %) than teenagers (12 %) and children (0 %). In bone fluorosis, the reported progression is adults > teenagers > children. The reported sequence is: adults > teenagers > children in bone fluorosis. The opposite tendency is seen with dental fluorosis: children > teens > adults. WHO (1984) found that fluoride concentrations over 10 mg/L increase the risk of debilitating skeletal fluorosis [61]. The current study found that the highest concentration was 3.80 mg/L, which reduces the likelihood of developing crippling skeletal fluorosis. The likelihood of developing this condition depends on a number of factors, including the amount of fluoride consumed, the type and solubility of the fluoride, the age of the individual at the first exposure, and their response to the fluoride [4,55].

**Table 5:** Comparative assessment of dental, bone and skeletal fluorosis.

Type of Fluorosis	Children	Teenagers	Adults
**Dental Fluorosis**	94 %	88 %	65 %
**Bone fluorosis**	0 %	12 %	59 %
**Skeletol Fluorosis**	0 %	0 %	0 %

**Table 6:** HQ (Fluoride) computation was done using deterministic approach in kasganj, U.P, India.

Location	HQ (Fluoride)		
	Children	Teenagers	Adults
S1	0.50	0.39	0.31
S2	1.21	0.93	0.75
S3	1.56	1.20	0.96
S4	1.56	1.20	0.96
S5	1.56	1.20	0.96
S6	2.08	1.60	1.28
S7	1.56	1.20	0.96
S8	2.25	1.73	1.39
S9	2.17	1.67	1.34
S10	2.41	1.85	1.49
S11	2.17	1.67	1.34
S12	1.94	1.49	1.20
S13	2.95	2.27	1.82
S14	2.10	1.61	1.29
S15	3.29	2.53	2.03
S16	2.30	1.77	1.42
S17	2.31	1.77	1.42

## 4 Assessment of fluoride effect on human health

### 4.1 Deterministic approach method

**Fig. 8:**
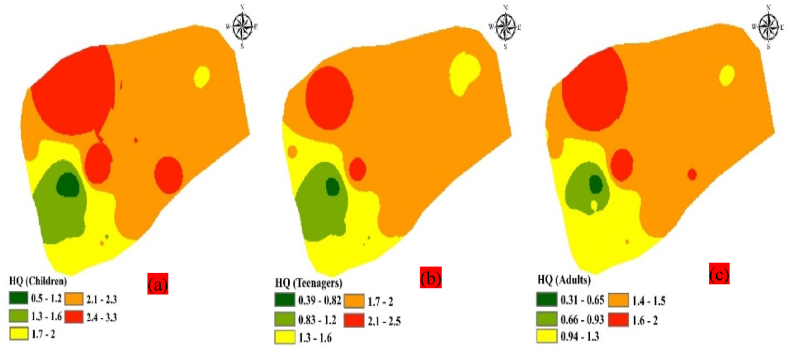
(a, b, c) Distribution of HQ (Fluoride) in targeted groups: a - children, b - teenagers and c - adults in sampled area of Kasganj, U. P, India.

This tool is a potent instrument utilized to assess the variables associated with measuring the danger to human health and to identify effective methods for managing those variables [34]. Using this technique, one can ascertain the effects of fluoride on the local resident's health [63]. Table 6 shows the computed data from the HQ research of toxins effects on targeted groups (children, teenagers, and adults), which was conducted using Eq. (2). Fig. 8 a, b, c displays the spatial distribution and dispersion of fluoride in three groups that were exposed, using the inverse distance weighting approach.

The calculation of oral exposure to fluoride was conducted using hazard quotients (HQ), which were stated in terms of mg/day and mg/kg/day, respectively. In the present study, HQ for targeted groups was calculated for the different locations of kasganj, U. P, India and it was found that there is a big difference in the exposure dose of different aged group people of sampled area. In the case of children (0.50–3.29), teenagers (0.39–2.53), and adults (0.31–2.03). Thus, the average concentration was found to be 1.99, 1.53 and 1.23 mg/kg/day respectively, while the maximum limit of the exposure dose of fluoride is found in area of Kasganj (3.29 mg/kg/day for children) as shown in Table 3 and Fig. 8 a, b, c. The range, however, surpassed the daily thresholds for Fluoride that were deemed "safe and acceptable" by the National Radiological Compensation Commission (2001) and USEPA standards [5,52,64]. According to the USEPA instructions, HQ ≥ 1 is not recommended as it leads to severe non-carcinogenic disease in the body.

### 4.2 Probability calculation using the MCS technique

**Fig. 9:**
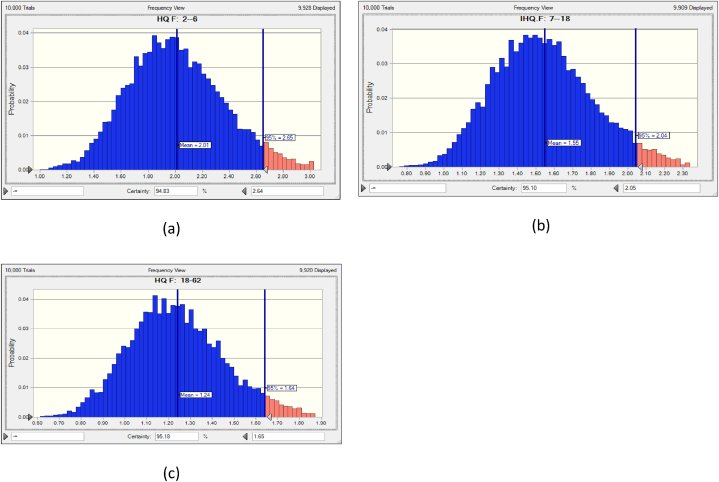
a, b, c Bar graphs displaying Fluoride HQ's uncertainty analysis.

The Monte Carlo simulation (MCS) approach was employed to calculate the HQ using Eq. (2). The probabilistic method of the MCS methodology incorporated F^−^ concentration, body weight, exposure frequency, inhalation rate, and other relevant data for all target groups [10,65]. The mathematical findings are displayed in Fig. 9 a, b, c for all populations that were subjected to exposure.

Exposures with HQ values greater than 1 are associated with an increased risk of non-cancer, long-term organ damage [10,11,65]. Based on the most likely calculations, children, teenagers, and adults have the highest HQ values. The 95th percentile HQ (fluoride) values for children, teenagers, and adults in Kasganj were 2.65, 2.01, and 1.64, respectively, as shown in Fig. 9 a, b, c. The 95th percentile of children's HQ values is 2.65, indicating high health risks. HRA has two main components: unpredictability and sensitivity, which operate independently but cannot be ignored. Lack of correct parameter information always causes unpredictability. MCS reduces health risk assessment uncertainty. Since the USEPA's values may vary by individual or place, risk evaluation typically is ambiguous. All parameter values are randomly selected in simulations to fix this. Sensitivity analysis focused on input variables and how they affected outcomes to assess uncertainty.

**Fig. 10:**
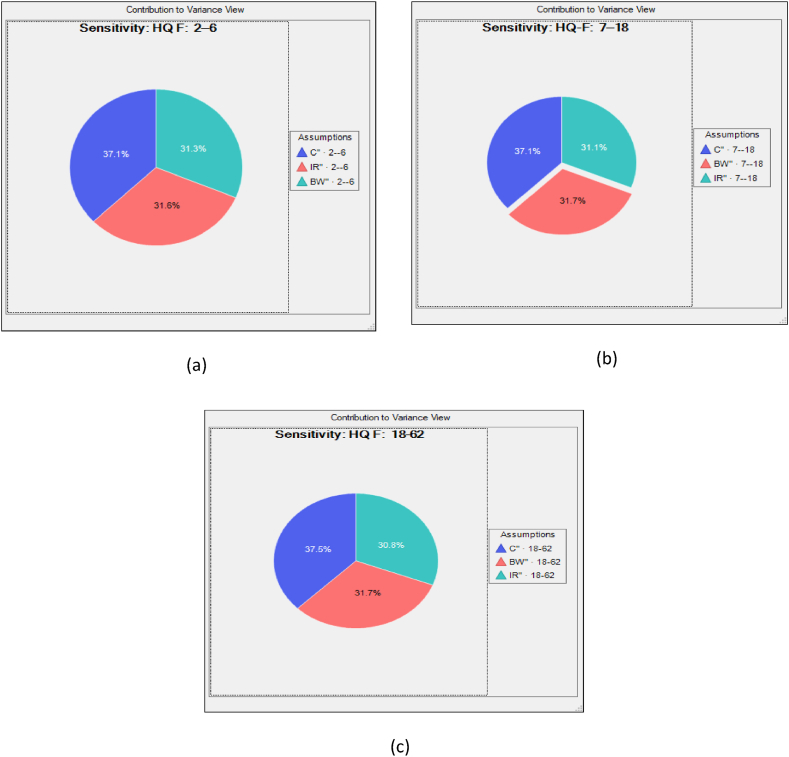
a, b, c Fluoride exposure sensitivity investigation.

The present research work is using to evaluate the possible health hazards by doing a sensitivity analysis on a range of input parameters, including C, IR, ED EF, BW and AT. The selected parameters were chosen at random to create tornado plots and do sensitivity analysis for the exposure groups i.e. children, teenagers and adults and found the descending order of C > BW > IR (fluoride) shown in Fig. 10 a, b, c. This model was used to calculate the HQ risk of drinking water, which includes non-cancerous contaminants. Metrics C and BW (fluoride) had the most impact on all target groups in Kasganj area. Their respective correlation coefficients ranged from 37.1 % to 37.5 % from 31.6 % to 31.7 % (F^−^). As the sensitivity analysis shows, the probability distributions of C and BW turned out to be crucial components in improving the accuracy of the outcomes.

### 4.3 HQ prediction using the ANN model

**Fig. 11:**
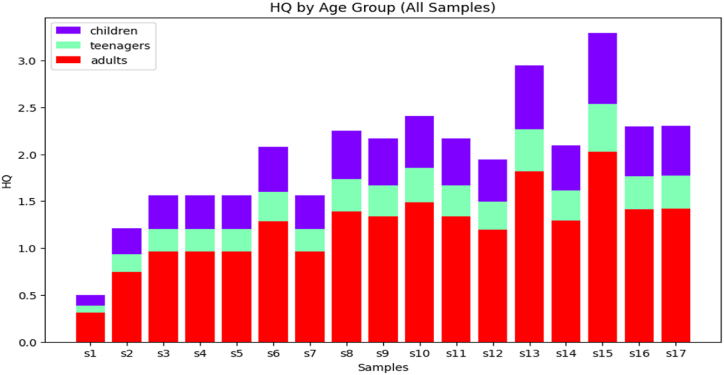
Variation of HQ (children, teenagers and adults) of Kasganj of various locations.

Fig. 11 depicts the variations of HQ in targeted groups in sampled area. The current study utilized a model that employed 80 % of the dataset for training purposes, while 10 % of the dataset was allocated for validation. Nevertheless, the remaining 10 % of the dataset has been employed for testing. The ANN model was then used to determine the HQ because of how well it works in this partitioning method. In order to estimate the HQ of the sampled area, the training results show that the ANN model with the best architecture out of five versions is the optimal combination.

**Fig. 12:**
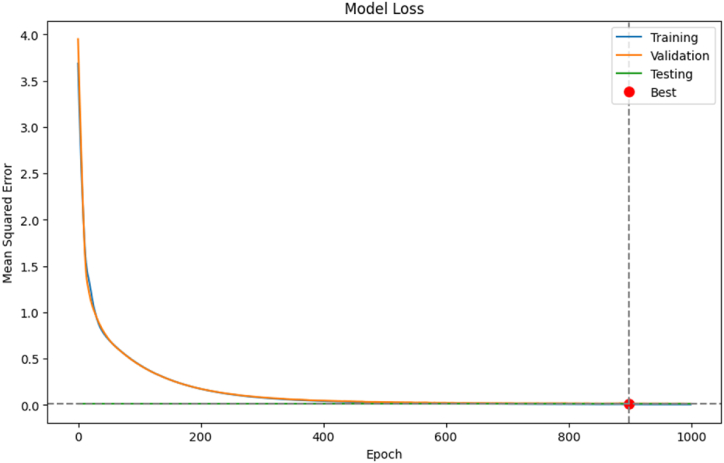
Training state plot of the ANN model.

The programs' great efficiency is confirmed by the large, positive, exceptionally high association (R^2^ = 99.89 %) between this network's training-phase prediction outputs and the actual outcomes. In line with the results of the regression correlation analysis, the ANN model's prediction results in testing showed an R^2^ value of 98.70 %, which is close to 100 %. This result highlights the model's well-specified nature and suggests that the ANN model with this architecture may account for nearly all of the reported variations in the atypical HQ values (R^2^ ≅ 1.00). As a result, out of total 85 samples observations were split into a training set of random 69 samples with cross-validation and testing fractions of 8 samples in each. The R^2^ value of 0.9989 in training and 0.9870 in testing while RMSE value in training and testing was 0.02230 and 0.0267. Fig. 12 shows the various stages of the ANN model. We partitioned the data samples for training, validation, and testing to find the best partition technique for system validation. In addition, the testing phase included data that had never been seen before, and the data was divided up using a random technique that picked out different fundamental points from the entire dataset. This process ensures that the ANN model is able to accurately forecast HQ. During testing, the method randomly chooses values for the test model from the entire dataset. The prediction accuracy displays the expected values at the evaluation state.

**Fig. 13:**
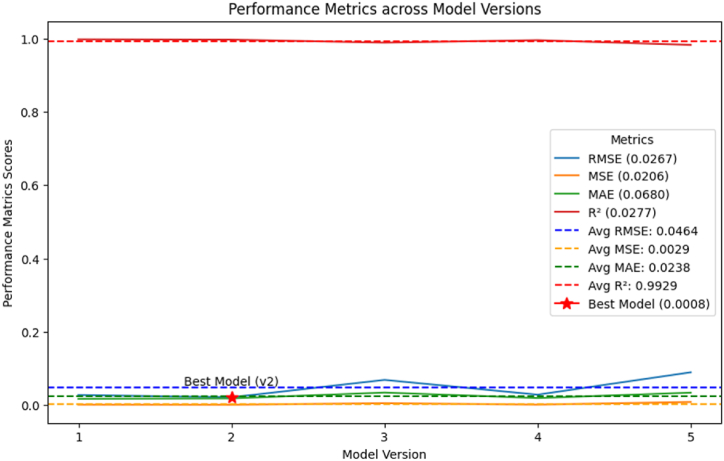
ANN model's performance during training.

The ANN model's performance is shown in Fig. 13. The best validation was learnt by the ANN model at epoch 530, with an MSE of 0.0004225. During the learning phase, the MSE of the ANN model's performance drastically decreased. A blue, green, and red line, respectively, represents the training process, the validation error, and the training error. Regular epoch counts indicate that there might be little errors in the training data; the training is stopped as soon as the validation error is removed.

**Fig. 14:**
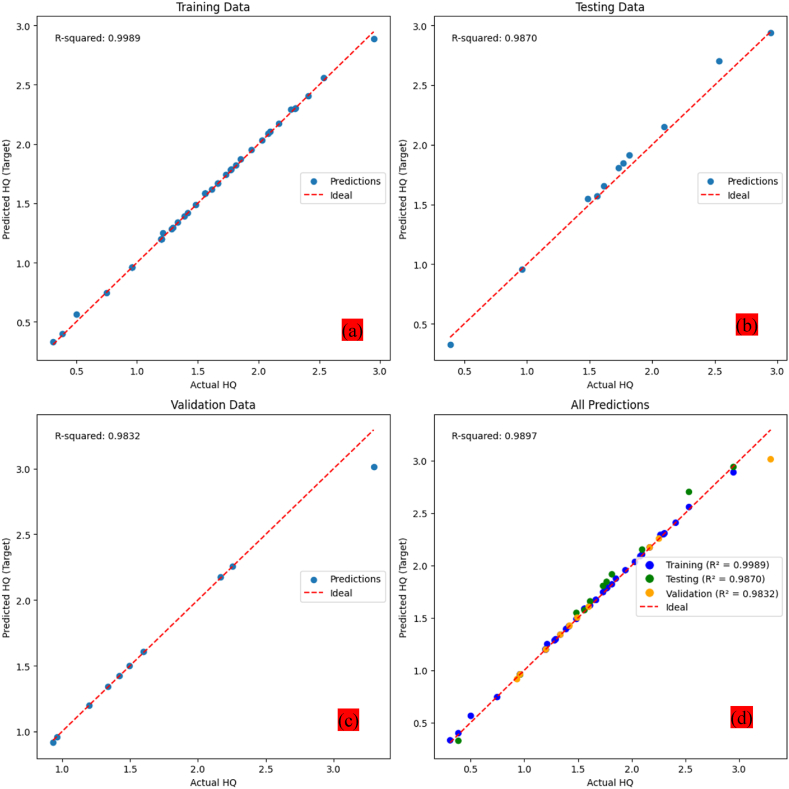
(a, b, c, d) Regression of ANN model during, a - Training, b - Testing, c - validation and d - overall.

Fig. 14 a, b, c,d depicts the performance of the best-trained ANN model on different datasets, by using a regression plot with the target values on the x-axis and the predicted output values on the y-axis. During training the points cluster closely around the regression line, and the R-squared value would be close to 1 indicating the model has learned the patterns in the training data effectively. During Validation, it generalizes to unseen data that was not used during training, indicates the model can apply its learned patterns to new data without overfitting (memorizing the training data too closely). And finally, during testing, it shows its performance on a completely independent dataset that it hasn't used before. It provides a final evaluation of the model's ability to generalize and make accurate predictions on new, unseen data.

## 5 Conclusion

The objective of this study was to investigate the fluoride concentration in drinking water sample, determine the potential dangers of fluoride using deterministic and probabilistic techniques, in the final predict the health risk by with the help of ANN in Kasganj district, Uttar Pradesh. Analysis of the results of the study reveals that the concentration of fluoride in groundwater sample of 15 regions of Kasganj district area higher than range recommended by BIS and WHO. The findings of the present study that the elevated fluoride concentrations present in the study area could potentially be attributed to the region's unique geological features, which render the water unfit for human consumption and unsuitable for other uses. Chronic daily intake of fluoride in adults consumes 1.20 times more fluoride than children and 1.5 times as much as teenagers, correspondingly. Children are more likely to have dental fluorosis with a probability of 94 %, than teenagers with a probability of 88 %, and adults with a probability of 65 %. However, adults with a probability of 59 % are more prone to bone fluorosis than teenagers with a probability of 12 % and children with a probability of 0 %. In the present study, the maximum concentration reported was 3.80 mg/L, diminishing the chances of developing crippling skeletal fluorosis. According to the health risk assessment, oral exposure due to the intake of contaminated groundwater can greatly affect the human health of that area. The Monte Carlo simulation emphasized high risks for children, which were consistent with high HQ values calculated. As the sensitivity analysis shows, the probability distributions of fluoride concentration and Body Weight turned out to be crucial components in improving the accuracy of the outcomes. The model performs exceptionally well with a high degree of accuracy with an overall R^2^ value of 98.97 % indicating that the model is highly effective. The categorical and age group analyses reveal meaningful insights, highlighting the model's ability to differentiate between various categories and age groups accurately.

## CRediT authorship contribution statement

**Raisul Islam:** Writing – original draft, Methodology, Investigation, Data curation, Conceptualization. **Alok Sinha:** Writing – original draft, Software. **Athar Hussain:** Writing – review & editing. **Mohammad Usama:** Writing – original draft, Software, Investigation. **Shahjad Ali:** Writing – original draft, Supervision, Methodology, Investigation, Conceptualization. **Salman Ahmed:** Writing – original draft, Methodology, Investigation. **Abdul Gani:** Writing – original draft, Software, Methodology, Investigation. **Najmaldin Ezaldin Hassan:** Writing – review & editing, Software. **Ali Akbar Mohammadi:** Writing – original draft, Methodology, Conceptualization. **Kamlesh Deshmukh:** Writing – original draft, Software, Investigation.

## Data availability statement

Data will be made available on request.

## Additional information

No additional information is available for this paper.

## Declaration of competing interest

The authors declare that they have no known competing financial interests or personal relationships that could have appeared to influence the work reported in this paper.

## References

[1] Khan M.A. (Jan. 2023). Potential health risk assessment, spatio-temporal hydrochemistry and groundwater quality of Yamuna river basin, Northern India. Chemosphere.

[2] Ali S., Mohammadi A.A., Ali H., Alinejad N., Maroosi M. (2022). Qualitative assessment of ground water using the water quality index from a part of Western Uttar Pradesh, North India. Desalin. Water Treat..

[3] Rao Q., Qiu Y., Li J. (Dec. 2019). Water quality assessment and variation trends analysis of the min river sea-entry section, China. Water, Air, Soil Pollut..

[4] Ali S., Kumari M., Gupta S.K., Sinha A., Mishra B.K. (2017). Investigation and mapping of fluoride-endemic areas and associated health risk—a case study of Agra, Uttar Pradesh, India. Hum. Ecol. Risk Assess..

[5] Ali S., Ali H., Pakdel M., Ghale Askari S., Mohammadi A.A., Rezania S. (Jan. 2022). Spatial analysis and probabilistic risk assessment of exposure to fluoride in drinking water using GIS and Monte Carlo simulation. Environ. Sci. Pollut. Res..

[6] Yadav K.K., Kumar V., Gupta N., Kumar S., Rezania S., Singh N. (Aug. 2019). Human health risk assessment: study of a population exposed to fluoride through groundwater of Agra city, India. Regul. Toxicol. Pharmacol..

[7] Alshammari F.R., Aljohani M., Botev L., O’malley L., Glenny A.M. (2021). Dental fluorosis prevalence in Saudi Arabia. Saudi Dent. J..

[8] Taylor P., Ayoob S., Gupta A.K. (August 2013. 2007). Critical Reviews in Environmental Science and Technology Fluoride in Drinking Water : A Review on the Status and Stress Effects Fluoride in Drinking Water : A Review.

[9] Dehghani M.H., Zarei A., Yousefi M., Baghal Asghari F., Haghighat G.A. (2019). Fluoride contamination in groundwater resources in the southern Iran and its related human health risks. Desalin. Water. Treat..

[10] Shahsavani S. (Jan. 2023). An ontology-based study on water quality: probabilistic risk assessment of exposure to fluoride and nitrate in Shiraz drinking water, Iran using fuzzy multi-criteria group decision-making models. Environ. Monit. Assess..

[11] Ali S. (2024). Geographical analysis of fluoride and nitrate and its probabilistic health risk assessment utilizing Monte Carlo simulation and GIS in potable water in rural areas of Mathura region, Uttar Pradesh, northern India. Heliyon.

[12] Yousefi Mahmood (2017). "Epidemiology of drinking water fluoride and its contribution to fertility, infertility, and abortion: an ecological study in West Azerbaijan Province, Poldasht County, Iran.". Fluoride.

[13] Yadav K.K., Gupta N., Kumar V., Khan S.A., Kumar A. (Feb. 2018). A review of emerging adsorbents and current demand for defluoridation of water: bright future in water sustainability. Environ. Int..

[14] Subba Rao N. (Dec. 2017). Controlling factors of fluoride in groundwater in a part of South India. Arab. J. Geosci..

[15] Maurya J., Pradhan S.N., Seema, Ghosh A.K. (May 2021). Evaluation of ground water quality and health risk assessment due to nitrate and fluoride in the Middle Indo-Gangetic plains of India. Hum. Ecol. Risk Assess. An Int. J..

[16] Ahada C.P.S., Suthar S. (Dec. 2019). Assessment of human health risk associated with high groundwater fluoride intake in southern districts of Punjab, India. Expo. Heal..

[17] Yousefi M., Ghoochani M., Hossein Mahvi A. (Feb. 2018). Health risk assessment to fluoride in drinking water of rural residents living in the Poldasht city, Northwest of Iran. Ecotoxicol. Environ. Saf..

[18] Fallahzadeh R.A. (Mar. 2018). Spatial variation and probabilistic risk assessment of exposure to fluoride in drinking water. Food Chem. Toxicol..

[19] Zhang L. (Jan. 2020). Spatial distribution of fluoride in drinking water and health risk assessment of children in typical fluorosis areas in north China. Chemosphere.

[20] Fluoride D., Systematic N.A., Choi A.L., Sun G., Zhang Y., Grandjean P. (2020). Review.

[21] Ali S., Gupta S.K., Sinha A., Khan S.U., Ali H. (2022). Health risk assessment due to fluoride contamination in groundwater of Bichpuri, Agra, India: a case study. Model. Earth Syst. Environ..

[22] Ali S. (2023). Variability of groundwater fluoride and its proportionate risk quantification via Monte Carlo simulation in rural and urban areas of Agra district, India. Sci. Rep..

[23] Mohammadi A.A., Ghaderpoori M., Yousefi M., Rahmatipoor M., Javan S. (2016). Prediction and modeling of fluoride concentrations in groundwater resources using an artificial neural network: a case study in Khaf. Environ. Heal. Eng. Manag..

[24] A. and prediction of groundwater using geospatial and ann Modeling (2010). Spatial prediction of fluoride concentration using artificial neural networks and geostatic models. Water Soil Sci.

[25] Nafouanti M.B., Li J., Mustapha N.A., Uwamungu P., AL-Alimi D. (Sep. 2021). Prediction on the fluoride contamination in groundwater at the Datong Basin, Northern China: comparison of random forest, logistic regression and artificial neural network. Appl. Geochemistry.

[26] Clesceri L.S., Greenberg A.E., Trussell R.R. (1990).

[27] EPA (1989).

[28] Bazeli J. (2020). Health risk assessment techniques to evaluate non-carcinogenic human health risk due to fluoride , nitrite and nitrate using Monte Carlo simulation and sensitivity analysis in Groundwater of Khaf County , Iran. Int. J. Environ. Anal. Chem..

[29] Erdal S., Buchanan S.N. (2005). A quantitative look at fluorosis, fluoride exposure, and intake in children using a health risk assessment approach. Environ. Health Perspect..

[30] Soleimani H. (2022). Groundwater quality evaluation and risk assessment of nitrate using Monte Carlo simulation and sensitivity analysis in rural areas of Divandarreh County, Kurdistan province, Iran. Int. J. Environ. Anal. Chem..

[31] Abbasnia A. (2019). Prediction of human exposure and health risk assessment to trihalomethanes in indoor swimming pools and risk reduction strategy. Hum. Ecol. Risk Assess. An Int. J..

[32] Rpa E.P.A. (1989).

[33] Soleimani H. (Sep. 2022). Probabilistic and deterministic approaches to estimation of non-carcinogenic human health risk due to heavy metals in groundwater resources of torbat heydariyeh, southeastern of Iran. Int. J. Environ. Anal. Chem..

[34] Soleimani H. (Aug. 2022). Groundwater quality evaluation and risk assessment of nitrate using Monte Carlo simulation and sensitivity analysis in rural areas of Divandarreh County, Kurdistan province, Iran. Int. J. Environ. Anal. Chem..

[35] Gazzaz N.M., Yusoff M.K., Aris A.Z., Juahir H., Ramli M.F. (Nov. 2012). Artificial neural network modeling of the water quality index for Kinta River (Malaysia) using water quality variables as predictors. Mar. Pollut. Bull..

[36] Al-Adhaileh M.H., Alsaade F.W. (2021). Kullanarak Su Kalitesinin Modellenmesi ve Tahminiyapay zeka - modelling and prediction of water quality by using artificial intelligence. Sustain. Times.

[37] Gani A., Singh M., Pathak S., Hussain A. (2023). Groundwater quality index development using the ANN model of Delhi Metropolitan City, India. Environ. Sci. Pollut. Res..

[38] Uddin M.G., Nash S., Mahammad Diganta M.T., Rahman A., Olbert A.I. (Nov. 2022). Robust machine learning algorithms for predicting coastal water quality index. J. Environ. Manage..

[39] Singh B., Sihag P., Singh V.P., Sepahvand A., Singh K. (Dec. 2021). Soft computing technique-based prediction of water quality index. Water Supply.

[40] Hanoon M.S. (Oct. 2021). Application of artificial intelligence models for modeling water quality in groundwater: comprehensive review, evaluation and future trends. Water, Air, Soil Pollut..

[41] Yaseen Z.M., Sulaiman S.O., Deo R.C., Chau K.-W. (Feb. 2019). An enhanced extreme learning machine model for river flow forecasting: state-of-the-art, practical applications in water resource engineering area and future research direction. J. Hydrol..

[42] BIS (2012). Indian standard drinking water specification (second revision). Bur. Indian Stand..

[43] WHO (2008). Guidelines for drinking-water quality: second addendum. World Heal. Organ. Press.

[44] Laxmankumar D., Satyanarayana E., Dhakate R., Saxena P.R. (Apr. 2019). Hydrogeochemical characteristics with respect to fluoride contamination in groundwater of Maheshwarm mandal, RR district, Telangana state, India. Groundw. Sustain. Dev..

[45] Duvva L.K., Panga K.K., Dhakate R., Himabindu V. (2022). Health risk assessment of nitrate and fluoride toxicity in groundwater contamination in the semi-arid area of Medchal, South India. Appl. Water Sci..

[46] Ahmed S., Khurshid S., Sultan W., Shadab M.B. (2020). Statistical analysis and water quality index development using gis of mathura city, Uttar Pradesh, India. Desalin. Water Treat..

[47] Ansari J.A., Umar R. (2019). Evaluation of hydrogeochemical characteristics and groundwater quality in the quaternary aquifers of Unnao District, Uttar Pradesh, India. HydroResearch.

[48] Chaurasia A.K., Pandey H.K., Tiwari S.K., Prakash R., Pandey P., Ram A. (Jul. 2018). Groundwater quality assessment using water quality index (WQI) in parts of varanasi district, Uttar Pradesh, India. J. Geol. Soc. India.

[49] Singh S. (2016). Fluoride contamination in groundwater in some villages of banda district , Uttar Pradesh. IJIRST –International J. Innov. Res. Sci. Technol..

[50] Dey S., Raju N.J. (2016). Geostatistical and Geospatial Approaches for the Characterization of Natural Resources in the Environment.

[51] Ali S. (2024). Groundwater quality assessment using water quality index and principal component analysis in the Achnera block, Agra district, Uttar Pradesh, Northern India. Sci. Rep..

[52] Carton R.J., Park A. (2006). Review of the 2006 United States national research council report.

[53] Naz A., Chowdhury A., Mishra B.K., Gupta S.K. (Oct. 2016). Metal pollution in water environment and the associated human health risk from drinking water: a case study of Sukinda chromite mine, India. Hum. Ecol. Risk Assess. An Int. J..

[54] (Apr. 1998). Pediatric death and fluoride-containing wheel cleaner. Ann. Emerg. Med..

[55] Ayoob S., Gupta A.K. (Dec. 2006). Fluoride in drinking water: a review on the status and stress effects. Crit. Rev. Environ. Sci. Technol..

[56] Meenakshi, Maheshwari R.C. (Sep. 2006). Fluoride in drinking water and its removal. J. Hazard Mater..

[57] World Health Organisation (2004).

[58] Choubisa S.L. (2001). Endemic fluorosis in southern Rajasthan, India. Fluoride.

[59] Fisher R.L. (Mar. 1989). Endemic fluorosis with spinal cord compression. Arch. Intern. Med..

[60] Maguire A., Zohouri F.V., Mathers J.C., Steen I.N., Hindmarch P.N., Moynihan P.J. (Nov. 2005). Bioavailability of fluoride in drinking water: a human experimental study. J. Dent. Res..

[61] WHO (1984). Fluorine and fluorides. IPCS International Programme on Chemical safety, Enviromental Health Criteria.

[62] Bharati P., Kubakaddi A., Rao M., Naik R.K. (Oct. 2005). Clinical symptoms of dental and skeletal fluorosis in gadag and bagalkot districts of Karnataka. J. Hum. Ecol..

[63] Gani A., Hussain A., Pathak S., Omar P.J. (Aug. 2024). Analysing heavy metal contamination in groundwater in the vicinity of Mumbai's landfill sites: an in-depth study. Top. Catal..

[64] Radfard M. (2019). "Health risk assessment to fluoride and nitrate in drinking water of rural residents living in the Bardaskan city, arid region, southeastern Iran. Desalin Water Treat.

[65] morovati R., Badeenezhad A., Najafi M., Azhdarpoor A. (Jan. 2024). Investigating the correlation between chemical parameters, risk assessment, and sensitivity analysis of fluoride and nitrate in regional groundwater sources using Monte Carlo. Environ. Geochem. Health.

